# 4,4′-(Propane-1,3-diyldi­oxy)dibenz­aldehyde

**DOI:** 10.1107/S1600536810021124

**Published:** 2010-06-09

**Authors:** Qamar Ali, Muhammad Raza Shah, Seik Weng Ng

**Affiliations:** a HEJ Research Institute of Chemistry, International Center for Chemical and Biological Sciences, University of Karachi, Karachi 75270, Pakistan; b Department of Chemistry, University of Malaya, 50603 Kuala Lumpur, Malaysia

## Abstract

The title compound, C_17_H_16_O_4_, is a dialdehyde in which two formyl­phen­oxy units are linked by a –CH_2_CH_2_CH_2_– chain; the mol­ecule is V-shaped with the middle methyl­ene C atom as the apex. The two benzene rings are aligned at 77.4 (1)°. In the crystal, mol­ecules are linked into centrosymmetric dimers by pairs of non-classical C—H⋯O hydrogen bonds.

## Related literature

For background to Schiff bases derived by condensing similar dialdehydes with primary amines, see: Zhang *et al.* (2008 ▶). For the crystal structure of the 2,2′-disubstituted analog, see: Hu *et al.* (2005 ▶).
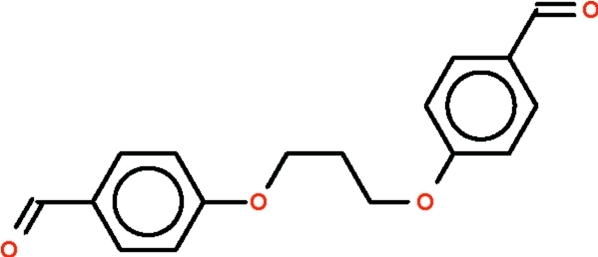


## Experimental

### 

#### Crystal data

C_17_H_16_O_4_
*M* *_r_* = 284.30
Monoclinic, 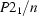

*a* = 15.3323 (15) Å
*b* = 4.6173 (5) Å
*c* = 20.2800 (19) Å
β = 104.783 (1)°
*V* = 1388.2 (2) Å^3^
*Z* = 4
Mo *K*α radiation
μ = 0.10 mm^−1^
*T* = 100 K
0.25 × 0.20 × 0.10 mm

#### Data collection

Bruker SMART APEXII diffractometer
8297 measured reflections
3113 independent reflections
2538 reflections with *I* > 2σ(*I*)
*R*_int_ = 0.027

#### Refinement

*R*[*F* ^2^ > 2σ(*F* ^2^)] = 0.039
*wR*(*F* ^2^) = 0.106
*S* = 1.03
3113 reflections
190 parameters
H-atom parameters constrained
Δρ_max_ = 0.25 e Å^−3^
Δρ_min_ = −0.22 e Å^−3^

### 

Data collection: *APEX2* (Bruker, 2009 ▶); cell refinement: *SAINT* (Bruker, 2009 ▶); data reduction: *SAINT*; program(s) used to solve structure: *SHELXS97* (Sheldrick, 2008 ▶); program(s) used to refine structure: *SHELXL97* (Sheldrick, 2008 ▶); molecular graphics: *X-SEED* (Barbour, 2001 ▶); software used to prepare material for publication: *publCIF* (Westrip, 2010 ▶).

## Supplementary Material

Crystal structure: contains datablocks global, I. DOI: 10.1107/S1600536810021124/ci5094sup1.cif

Structure factors: contains datablocks I. DOI: 10.1107/S1600536810021124/ci5094Isup2.hkl

Additional supplementary materials:  crystallographic information; 3D view; checkCIF report

## Figures and Tables

**Table 1:** Hydrogen-bond geometry (Å, °)

*D*—H⋯*A*	*D*—H	H⋯*A*	*D*⋯*A*	*D*—H⋯*A*
C16—H16⋯O1^i^	0.95	2.41	3.287 (2)	154

Symmetry code: (i) 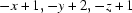.

**Fig. 1.**
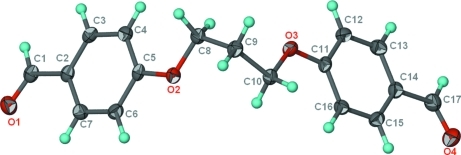
Displacement ellipsoid plot (Barbour, 2001) of C17H16O4 at the 70% probability level; H atoms are drawn as spheres of arbitrary radius.

## supplementary crystallographic information

### Comment

The two-arm aldehyde is intended for condensation with primary amines to form Schiff bases, which, in a subsequent step, will be reacted with *β*-cyclodextrin to furnish inclusion compounds. The idea for this theme draws on a report on such compounds of poly(Schiff bases) (Zhang *et al.*, 2008). The flexibilty of the Schiff base can be controlled by varying the position of the formyl group; the title compound has the the formyl groups in the 4,4'-positions. The crystal structure of the 2,2'-substituted compound has been reported (Hu *et al.*, 2005). The molecule of C_17_H_16_O_4_ (Scheme I) is V-shaped with the middle methylene carbon as the apex (Fig. 1).

### Experimental

4-Hydroxybenzaldehyde (1 g, 8.2 mmol) was dissolved in acetone (25 ml). To the solution was added potassium carbonate (2.3 g, 16.4 mmol). The mixture was heated for 1 h. 1,3-Dibromopropane (0.29 ml, 2.7 mmol) was added and the mixture heated for another hour. The mixture was set aside for 8 h. The solvent was removed and the solid material was extracted with ethyl acetate. The solvent was again removed and the product purified by column chromatography by using dichloromethane-hexane (1:4) as mobile phase. Single crystals were obtained by recrystallization from dichloromethane.

### Refinement

H atoms were placed in calculated positions [C–H = 0.95–0.99 Å] and were included in the refinement in the riding model approximation, with *U_iso_*(H) = 1.2*U_eq_*(C).

### Crystal data

**Table:**

C_17_H_16_O_4_	*F*(000) = 600
*M**_r_* = 284.30	*D*_x_ = 1.360 Mg m^−^^3^
Monoclinic, *P*2_1_/*n*	Mo *K*α radiation, λ = 0.71073 Å
Hall symbol: -P 2yn	Cell parameters from 2677 reflections
*a* = 15.3323 (15) Å	θ = 3.1–28.0°
*b* = 4.6173 (5) Å	µ = 0.10 mm^−^^1^
*c* = 20.2800 (19) Å	*T* = 100 K
β = 104.783 (1)°	Plate, colourless
*V* = 1388.2 (2) Å^3^	0.25 × 0.20 × 0.10 mm
*Z* = 4	

### Data collection

**Table:**

Bruker SMART APEXII diffractometer	2538 reflections with *I* > 2σ(*I*)
Radiation source: fine-focus sealed tube	*R*_int_ = 0.027
graphite	θ_max_ = 27.5°, θ_min_ = 1.5°
ω scans	*h* = −19→19
8297 measured reflections	*k* = −5→5
3113 independent reflections	*l* = −19→26

### Refinement

**Table:**

Refinement on *F*^2^	Primary atom site location: structure-invariant direct methods
Least-squares matrix: full	Secondary atom site location: difference Fourier map
*R*[*F*^2^ > 2σ(*F*^2^)] = 0.039	Hydrogen site location: inferred from neighbouring sites
*wR*(*F*^2^) = 0.106	H-atom parameters constrained
*S* = 1.02	*w* = 1/[σ^2^(*F*_o_^2^) + (0.0513*P*)^2^ + 0.4617*P*] where *P* = (*F*_o_^2^ + 2*F*_c_^2^)/3
3113 reflections	(Δ/σ)_max_ = 0.001
190 parameters	Δρ_max_ = 0.25 e Å^−^^3^
0 restraints	Δρ_min_ = −0.21 e Å^−^^3^

### Fractional atomic coordinates and isotropic or equivalent isotropic displacement parameters (Å^2^)

**Table:**

	*x*	*y*	*z*	*U*_iso_*/*U*_eq_	
O1	0.33791 (6)	1.4272 (2)	0.27971 (5)	0.0247 (2)	
O2	0.37445 (6)	0.5332 (2)	0.52487 (5)	0.0191 (2)	
O3	0.36450 (6)	0.5695 (2)	0.70403 (5)	0.0191 (2)	
O4	0.58320 (6)	1.2514 (2)	0.97043 (5)	0.0290 (3)	
C1	0.28543 (9)	1.3387 (3)	0.31097 (7)	0.0193 (3)	
H1	0.2260	1.4154	0.2992	0.023*	
C2	0.30737 (8)	1.1217 (3)	0.36563 (7)	0.0171 (3)	
C3	0.24107 (8)	1.0286 (3)	0.39613 (7)	0.0183 (3)	
H3	0.1816	1.1028	0.3805	0.022*	
C4	0.25983 (8)	0.8289 (3)	0.44913 (7)	0.0182 (3)	
H4	0.2137	0.7657	0.4694	0.022*	
C5	0.34744 (8)	0.7227 (3)	0.47213 (6)	0.0166 (3)	
C6	0.41484 (8)	0.8113 (3)	0.44128 (7)	0.0188 (3)	
H6	0.4742	0.7361	0.4566	0.023*	
C7	0.39463 (9)	1.0082 (3)	0.38860 (7)	0.0192 (3)	
H7	0.4404	1.0678	0.3676	0.023*	
C8	0.30783 (8)	0.4333 (3)	0.55834 (7)	0.0182 (3)	
H8A	0.2590	0.3283	0.5257	0.022*	
H8B	0.2810	0.5991	0.5771	0.022*	
C9	0.35588 (9)	0.2331 (3)	0.61526 (7)	0.0190 (3)	
H9A	0.3901	0.0869	0.5964	0.023*	
H9B	0.3103	0.1290	0.6331	0.023*	
C10	0.42019 (8)	0.3892 (3)	0.67370 (7)	0.0176 (3)	
H10A	0.4544	0.2486	0.7074	0.021*	
H10B	0.4635	0.5084	0.6567	0.021*	
C11	0.40426 (8)	0.7236 (3)	0.76107 (6)	0.0164 (3)	
C12	0.34516 (8)	0.8830 (3)	0.78886 (7)	0.0189 (3)	
H12	0.2821	0.8762	0.7685	0.023*	
C13	0.37858 (9)	1.0507 (3)	0.84602 (7)	0.0202 (3)	
H13	0.3383	1.1611	0.8647	0.024*	
C14	0.47139 (8)	1.0595 (3)	0.87679 (7)	0.0187 (3)	
C15	0.52930 (8)	0.8986 (3)	0.84844 (7)	0.0189 (3)	
H15	0.5923	0.9038	0.8691	0.023*	
C16	0.49718 (8)	0.7308 (3)	0.79069 (7)	0.0174 (3)	
H16	0.5375	0.6227	0.7716	0.021*	
C17	0.50495 (9)	1.2379 (3)	0.93787 (7)	0.0235 (3)	
H17	0.4622	1.3516	0.9531	0.028*	

### Atomic displacement parameters (Å^2^)

**Table:**

	*U*^11^	*U*^22^	*U*^33^	*U*^12^	*U*^13^	*U*^23^
O1	0.0269 (5)	0.0271 (5)	0.0213 (5)	0.0011 (4)	0.0081 (4)	0.0041 (4)
O2	0.0179 (4)	0.0231 (5)	0.0158 (5)	0.0023 (4)	0.0036 (4)	0.0036 (4)
O3	0.0165 (4)	0.0234 (5)	0.0163 (5)	0.0004 (4)	0.0020 (3)	−0.0030 (4)
O4	0.0248 (5)	0.0375 (6)	0.0228 (6)	−0.0053 (4)	0.0027 (4)	−0.0083 (5)
C1	0.0220 (6)	0.0177 (6)	0.0168 (7)	0.0004 (5)	0.0027 (5)	−0.0033 (5)
C2	0.0200 (6)	0.0162 (6)	0.0139 (6)	−0.0002 (5)	0.0020 (5)	−0.0036 (5)
C3	0.0168 (6)	0.0190 (6)	0.0169 (7)	0.0015 (5)	0.0006 (5)	−0.0027 (5)
C4	0.0171 (6)	0.0207 (6)	0.0165 (7)	−0.0013 (5)	0.0039 (5)	−0.0019 (5)
C5	0.0197 (6)	0.0166 (6)	0.0118 (6)	0.0005 (5)	0.0010 (5)	−0.0027 (5)
C6	0.0166 (6)	0.0212 (7)	0.0177 (7)	0.0022 (5)	0.0026 (5)	−0.0018 (5)
C7	0.0189 (6)	0.0211 (7)	0.0180 (7)	−0.0012 (5)	0.0055 (5)	−0.0017 (5)
C8	0.0187 (6)	0.0199 (7)	0.0158 (7)	−0.0021 (5)	0.0039 (5)	−0.0018 (5)
C9	0.0208 (6)	0.0174 (6)	0.0181 (7)	−0.0019 (5)	0.0035 (5)	−0.0009 (5)
C10	0.0184 (6)	0.0182 (6)	0.0160 (7)	0.0010 (5)	0.0038 (5)	−0.0005 (5)
C11	0.0191 (6)	0.0155 (6)	0.0135 (6)	−0.0013 (5)	0.0022 (5)	0.0025 (5)
C12	0.0154 (6)	0.0218 (7)	0.0192 (7)	−0.0006 (5)	0.0037 (5)	0.0012 (5)
C13	0.0194 (6)	0.0205 (7)	0.0221 (7)	0.0011 (5)	0.0077 (5)	−0.0014 (6)
C14	0.0205 (6)	0.0190 (6)	0.0163 (7)	−0.0018 (5)	0.0037 (5)	0.0006 (5)
C15	0.0160 (6)	0.0204 (7)	0.0186 (7)	−0.0006 (5)	0.0015 (5)	0.0027 (5)
C16	0.0165 (6)	0.0190 (6)	0.0173 (7)	0.0016 (5)	0.0053 (5)	0.0009 (5)
C17	0.0242 (7)	0.0258 (7)	0.0214 (7)	−0.0022 (6)	0.0073 (5)	−0.0026 (6)

### Geometric parameters (Å, °)

**Table:**

O1—C1	1.2152 (16)	C8—H8A	0.99
O2—C5	1.3620 (15)	C8—H8B	0.99
O2—C8	1.4389 (15)	C9—C10	1.5167 (17)
O3—C11	1.3623 (15)	C9—H9A	0.99
O3—C10	1.4382 (15)	C9—H9B	0.99
O4—C17	1.2145 (17)	C10—H10A	0.99
C1—C2	1.4682 (19)	C10—H10B	0.99
C1—H1	0.95	C11—C12	1.3931 (18)
C2—C3	1.3863 (18)	C11—C16	1.3981 (17)
C2—C7	1.4014 (18)	C12—C13	1.3796 (19)
C3—C4	1.3894 (19)	C12—H12	0.95
C3—H3	0.95	C13—C14	1.4009 (17)
C4—C5	1.3938 (18)	C13—H13	0.95
C4—H4	0.95	C14—C15	1.3895 (19)
C5—C6	1.3989 (18)	C14—C17	1.4674 (19)
C6—C7	1.3765 (19)	C15—C16	1.3859 (18)
C6—H6	0.95	C15—H15	0.95
C7—H7	0.95	C16—H16	0.95
C8—C9	1.5153 (18)	C17—H17	0.95
			
C5—O2—C8	117.78 (10)	C10—C9—H9A	108.9
C11—O3—C10	118.66 (9)	C8—C9—H9B	108.9
O1—C1—C2	124.65 (12)	C10—C9—H9B	108.9
O1—C1—H1	117.7	H9A—C9—H9B	107.7
C2—C1—H1	117.7	O3—C10—C9	105.71 (10)
C3—C2—C7	118.83 (12)	O3—C10—H10A	110.6
C3—C2—C1	119.72 (11)	C9—C10—H10A	110.6
C7—C2—C1	121.44 (12)	O3—C10—H10B	110.6
C2—C3—C4	121.36 (12)	C9—C10—H10B	110.6
C2—C3—H3	119.3	H10A—C10—H10B	108.7
C4—C3—H3	119.3	O3—C11—C12	115.02 (11)
C3—C4—C5	118.95 (12)	O3—C11—C16	124.28 (11)
C3—C4—H4	120.5	C12—C11—C16	120.69 (12)
C5—C4—H4	120.5	C13—C12—C11	119.74 (11)
O2—C5—C4	124.25 (12)	C13—C12—H12	120.1
O2—C5—C6	115.34 (11)	C11—C12—H12	120.1
C4—C5—C6	120.40 (12)	C12—C13—C14	120.51 (12)
C7—C6—C5	119.66 (12)	C12—C13—H13	119.7
C7—C6—H6	120.2	C14—C13—H13	119.7
C5—C6—H6	120.2	C15—C14—C13	118.95 (12)
C6—C7—C2	120.77 (12)	C15—C14—C17	121.73 (12)
C6—C7—H7	119.6	C13—C14—C17	119.32 (12)
C2—C7—H7	119.6	C16—C15—C14	121.44 (12)
O2—C8—C9	106.81 (10)	C16—C15—H15	119.3
O2—C8—H8A	110.4	C14—C15—H15	119.3
C9—C8—H8A	110.4	C15—C16—C11	118.67 (12)
O2—C8—H8B	110.4	C15—C16—H16	120.7
C9—C8—H8B	110.4	C11—C16—H16	120.7
H8A—C8—H8B	108.6	O4—C17—C14	124.94 (13)
C8—C9—C10	113.45 (11)	O4—C17—H17	117.5
C8—C9—H9A	108.9	C14—C17—H17	117.5
			
O1—C1—C2—C3	−177.57 (13)	C11—O3—C10—C9	176.03 (10)
O1—C1—C2—C7	3.3 (2)	C8—C9—C10—O3	65.84 (13)
C7—C2—C3—C4	0.75 (19)	C10—O3—C11—C12	−177.41 (11)
C1—C2—C3—C4	−178.41 (12)	C10—O3—C11—C16	3.47 (18)
C2—C3—C4—C5	0.48 (19)	O3—C11—C12—C13	−178.89 (12)
C8—O2—C5—C4	0.89 (18)	C16—C11—C12—C13	0.26 (19)
C8—O2—C5—C6	−179.69 (11)	C11—C12—C13—C14	−0.7 (2)
C3—C4—C5—O2	178.02 (12)	C12—C13—C14—C15	0.6 (2)
C3—C4—C5—C6	−1.37 (19)	C12—C13—C14—C17	−179.52 (13)
O2—C5—C6—C7	−178.41 (11)	C13—C14—C15—C16	0.0 (2)
C4—C5—C6—C7	1.0 (2)	C17—C14—C15—C16	−179.92 (12)
C5—C6—C7—C2	0.2 (2)	C14—C15—C16—C11	−0.42 (19)
C3—C2—C7—C6	−1.1 (2)	O3—C11—C16—C15	179.37 (12)
C1—C2—C7—C6	178.04 (12)	C12—C11—C16—C15	0.29 (19)
C5—O2—C8—C9	−178.76 (10)	C15—C14—C17—O4	−3.7 (2)
O2—C8—C9—C10	70.22 (13)	C13—C14—C17—O4	176.44 (14)

### Hydrogen-bond geometry (Å, °)

**Table:**

*D*—H···*A*	*D*—H	H···*A*	*D*···*A*	*D*—H···*A*
C6—H6···O2^i^	0.95	2.57	3.508 (2)	168
C16—H16···O1^ii^	0.95	2.41	3.287 (2)	154

Symmetry codes: (i) −*x*+1, −*y*+1, −*z*+1; (ii) −*x*+1, −*y*+2, −*z*+1.
